# Gestational diabetes mellitus: challenges in diagnosis and management

**DOI:** 10.1186/s40200-015-0169-7

**Published:** 2015-05-12

**Authors:** Bonaventura C. T. Mpondo, Alex Ernest, Hannah E. Dee

**Affiliations:** School of Medicine and Dentistry, College of Health Sciences, University of Dodoma, Dodoma, Tanzania; Department of Internal Medicine, College of Health Sciences, Dodoma, Tanzania; Department of Obstetrics and Gynaecology, College of Health Sciences, PO Box 395, Dodoma, Tanzania; Weill Cornell Medical College, New York, USA

**Keywords:** Gestation diabetes mellitus, Glucose intolerance, Screening, Glycaemic control, Insulin, Oral agents

## Abstract

Gestational diabetes mellitus (GDM) is a well-characterized disease affecting a significant population of pregnant women worldwide. It has been widely linked to undue weight gain associated with factors such as diet, obesity, family history, and ethnicity. Poorly controlled GDM results in maternal and fetal morbidity and mortality. Improved outcomes therefore rely on early diagnosis and tight glycaemic control. While straightforward protocols exist for screening and management of diabetes mellitus in the general population, management of GDM remains controversial with conflicting guidelines and treatment protocols. This review highlights the diagnostic and management options for GDM in light of recent advances in care.

## Introduction

Gestational diabetes mellitus (GDM), by definition, is any degree of glucose intolerance with onset or first recognition during pregnancy [1, 2]. This definition applies regardless of whether treatment involves insulin or diet modification alone; it may also apply to conditions that persist after pregnancy. GDM affects roughly 7 % of pregnancies with an incidence of more than 200,000 cases per year [2]. The prevalence, however, varies from 1–14 %, depending on the population and the diagnostic criteria that have been used [2].

GDM is the most common cause of diabetes during pregnancy, accounting for up to 90 % of pregnancies complicated by diabetes [2]. Women with GDM have a 40–60 % chance of developing diabetes mellitus over the 5–10 years after pregnancy [3].

Although GDM has been recognized as a disease for some time, it remains a controversial entity with conflicting guidelines and treatment protocols.

## Review

### Screening

The first screening test for GDM, proposed in 1973, consisted of the 1-h 50 gm oral glucose tolerance test [4]. While some guidelines recommend universal screening, others exempt those patients who are categorized as low-risk. Evidence suggests that universal screening improves pregnancy outcomes compared to selective screening [5]. However, other researchers argue that screening women based on their clinical characteristics allows for more efficient selective screening for GDM [6].

Low-risk patients include those women with the following characteristics: <25 years of age; normal body weight; no first-degree relatives with diabetes; no history of abnormal glucose metabolism; no history of poor obstetric outcomes; and not from an ethnic group with a high diabetes prevalence (Hispanic American, Native American, Asian American, African American, and Pacific Islander) [7, 8]. Although some experts recommend against screening these low-risk patients routinely [2], selective screening could miss approximately 4 % of patients with GDM [9].

Pregnant women with factors conferring a high risk of GDM (marked obesity, previous history of GDM, glycosuria, or family history of diabetes) should be screened for GDM as soon as possible, preferably during their first antenatal visit. If negative, they should be retested at the beginning of their third trimester between 24 to 28 weeks of gestation. Women who are categorized as average risk (neither high nor low risk) should also be screened between 24 and 28 weeks of gestation [2]. When universal screening is implemented, patients with no recognized risk factors for GDM also undergo a 1-h glucose challenge test at 24 to 28 weeks of pregnancy. The classification criteria are summarized in Table 1 [6].

**Table 1:** Categorizing groups at risk for gestation diabetes mellitus

Risk category	Clinical characteristics
High risk	• Marked obesity
	• Diabetes in first degree relative
	• Current glycosuria
	• Previous history of GDM or glucose intolerance
	• Previous poor obstetric outcome (e.g. an infant with marosomia)
Average risk	• Neither high nor low risk
Low risk	• Age <25 years
	• No history of poor obstetric outcomes
	• Belongs to low risk ethnic groups (ethnic groups other than Hispanic, African American, Native American, South Asian, East Asian, Pacific Islander, or Indigenous Australian)
	• No diabetes in first degree relative
	• No history of abnormal glucose tolerance
	• Normal pre-pregnancy weight and pregnancy weight gain

Fasting plasma glucose and postprandial plasma glucose have been shown to have low sensitivity as screening tests for GDM [10, 11], and therefore they are not recommended for screening.

In general, there are two approaches to the evaluation of women for GDM: the one-step approach and the two-step approach. In the one-step approach, a diagnostic oral glucose tolerance test (OGTT) is performed without prior plasma or serum glucose screening. This approach may be cost effective in high-risk patients. In the two-step approach, initial screening involves the glucose challenge test, which measures the plasma or serum glucose concentration 1 h after a 50-gm oral glucose load. The diagnostic oral glucose challenge test is performed only in the subset of women found to have plasma or serum glucose concentration values exceeding the threshold for the glucose challenge test.

When the threshold for glucose challenge test is >140 mg/dl (7.8 mmol/l), the sensitivity is 80 %; when it is 130 mg/dl (7.2 mmol/l), the sensitivity becomes 90 % [1]. Whichever approach is used, the diagnosis of GDM is established only after performing an OGTT.

### Diagnostic criteria

There are two major diagnostic criteria for the 3-h 100-gm OGTT used in the United States: the Carpenter-Coustan criteria and the National Diabetes Data Group (NDDG) criteria. The Carpenter-Coustan criteria derive from the work of O’Sullivan and Mahan [4], which Carpenter and Coustan modified in 1982 [12]. In this method, diagnosis of GDM is based on exceeding two or more of the following threshold values:

- Fasting serum glucose concentration of 95 mg/dl (5.3 mmol/l)
- 1-h serum glucose concentration of 180 mg/dl (10.0 mmol/l)
- 2-h serum glucose concentration of 155 mg/dl (8.6 mmol/l)
- 3-h serum glucose concentration of 140 mg/dl (7.8 mmol/l)

The NDDG criteria, meanwhile, are slightly less inclusive than the Carpenter-Coustan criteria [13]. Furthermore, the NDDG criteria were found to be less sensitive in diagnosing GDM and in predicting incidence of perinatal morbidities [14]. The NDDG criteria are also based on exceeding two or more of the threshold values, which are as follows:

- Fasting serum glucose concentration of 105 mg/dl
- 1-h serum glucose concentration of 190 mg/dl
- 2-h serum glucose concentration of 165 mg/dl
- 3-h serum glucose concentration of 145 mg/dl

Alternatively, the American Diabetes (ADA) criteria for GDM diagnosis rely on a 75-gm glucose load and consider fasting serum glucose concentration, 1-h glucose concentration, and 2-h glucose concentration [15]. The glucose threshold values are, respectively, 95 mg/dl (5.3 mmol/l), 180 mg/dl (10.0 mmol/l), and 155 mg/dl (8.6 mmol/l). Again, two or more abnormal values are required for diagnosis. Although these major criteria all require two or more abnormal values for diagnosis, studies have shown that a single abnormal value is significantly associated with increased risk of perinatal morbidities [16].

The World Health Organization (WHO) recommends using a 75-gm glucose tolerance test for screening and diagnosis. The threshold values are a fasting glucose concentration of more than 126 mg/dl (7.0 mmol/l) and/or a 2-h glucose concentration of more than 140 mg/dl (7.8 mmol/l) [17]. When the WHO criteria are used, approximately twice as many patients will be diagnosed with GDM compared to other criteria. However, there is no proven additional clinical benefit with the use of WHO criteria [18]. The criteria for diagnosis of GDM are summarized in Table 2.

**Table 2:** Diagnostic criteria for gestation diabetes mellitus with their respective glucose values

Diagnostic criteria	Fasting (mg/dl [mmol/l])	1-h (mg/dl [mmol/l])	2-h (mg/dl [mmol/l])	3-h (mg/dl [mmol/l])
100-gm OGTT Carpenter/Coustan (two or more abnormal)	95 (5.3)	180 (10.0)	155 (8.6)	140 (7.8)
100-gm OGTT NDDG (two or more abnormal)	105 (5.8)	190 (10.6)	165 (9.2)	145 (8.1)
75-gm OGTT WHO (one or more abnormal)	92-125 (5.1-6.9)	≥180 (10.0)	153-199 (8.5-11.0)	-
75-gm OGTT ADA	95 (5.3)	180 (10.0)	155 (8.6)	-

OGTT = Oral glucose tolerance test, NDDG = National Diabetes Data Group, WHO = World Health Organization 2013, ADA = American Diabetes Association

### Treatment

Evidence shows that screening for and treating GDM lead to the reduction of perinatal morbidity and the improvement of post-delivery outcomes [19]. As in other types of diabetes, the cornerstone of GDM management is glycaemic control [1]. Glycaemic control has been shown to reduce adverse outcomes in pregnant women with GDM [20, 21].

### Target glucose values

Experts recommend that women with GDM should maintain the following capillary blood glucose values: preprandial glucose <95 mg/dl (5.3 mmol/l), 1-h postprandial glucose <140 mg/dl (7.8 mmol/l), and 2-h postprandial glucose <120 mg/dl (6.7 mmol/l) [1]. The American College of Obstetrics and Gynaecology (ACOG) has similar guidelines, the only exception being that both 130 mg/dl and 140 mg/dl 1-h postprandial glucose values are considered acceptable [22]. Other recommendations suggest maintaining fasting glucose levels of <90–99 mg/dl (5.0–5.5 mmol/l), 1-h postprandial glucose levels of <140 mg/dl (7.8 mmol/l), and 2-h postprandial glucose levels of <120–127 mg/dl (6.7–7.1 mmol/l) [23].

Even if it is not possible to achieve the recommended levels of glycaemic control, any improvement can be beneficial given that perinatal complications are linked to increasing serum glucose values [21, 24]. Despite the benefits of glycaemic control, however, studies have shown that very low target glucose values (<87 mg/dl) are associated with increased rates of intrauterine fetal growth retardation [20].

### Medical nutrition therapy (MNT)

The first line of management for women with gestational diabetes mellitus is dietary modification, often called medical nutrition therapy [25]. Evidences indicates that nutrition therapy is effective in reducing pregnancy and perinatal complications and also in attaining glycaemic control [25].

According to ADA recommendations, carbohydrate intake should be approximately 40 % of total calorie intake and should be selected from foods with low glycaemic index values [26]. In pregnant women of normal body weight (BMI between 18.5–24.9), the recommendation is to consume 30–32 kcal/kg body weight, especially during the second half of pregnancy [27]. However, those who are overweight (BMI of 25 to 29.9) should ingest approximately 25 kcal/kg body weight [28]. Other guidelines recommend caloric intake based on BMI as follows: 30 kcal/kg for a BMI of 22–25, 24 kcal/kg for a BMI of 26–29, and 12–15 kcal/kg for a BMI of >30.

75–80 % of women with GDM become euglycaemic by following these caloric distribution guidelines. Assessing fasting ketonuria provides a method of confirming a woman’s caloric restriction, because caloric restriction of at least 50 % has been associated with ketogenesis [29]. On the other hand, moderate caloric restriction of about 33 % has been associated with controlled glucose levels without elevation of free fatty acids and ketonaemia [28, 29]. Caloric restriction should be approached cautiously, because studies show that elevated maternal ketone levels are associated with impaired psychomotor development [30].

Compared to diet alone, exercise with dietary modifications has been found to lead to improved glycaemic control in one study [31]. The proposed mechanism for such an improvement in glycaemic control is heightened sensitivity of peripheral tissues to insulin. A supervised home-based cycling program was helpful in maintaining normal postprandial glucose levels in pregnant women with diet-controlled GDM [32]. That said, another trial using a partially home-based exercise program found no reduction in blood glucose level [33]. This cohort did demonstrate improved cardiovascular fitness, however.

Based on the available evidence on the benefits of exercise in managing GDM, ADA recommends moderate exercise programs for women without medical or obstetrical complications [15]. There are no specific guidelines, however, on how to employ exercise regimes to achieve glycaemic control. For the general population, experts tend to recommend exercising 3 or more times a week for about 30 min.

### Pharmacotherapy

Pharmacological intervention in the management of GDM is usually employed when women fail to meet established goals with conventional therapy of diet and exercise. It is also indicated when elevated fasting glucose levels occur while on conventional therapy, because dietary modification has limited effect on these levels. Although most women achieve adequate glycaemic control with conventional therapy, 30–40 % do require the addition of pharmacologic therapy at some point during their pregnancies [34]. The pharmacological options in this case include insulin or oral hypoglycaemic agents (metformin and glyburide) [35, 36].

#### Insulin

Insulin therapy is the most commonly used pharmacotherapy once MNT fails to achieve desired outcomes. Insulin regimens often include intermediate-acting insulins such as isophane and short-acting agents such as regular recombinant insulin (Humulin R). Pharmacotherapy can also involve the insulin analogues aspart and lisipro. Insulin therapy decreases the frequency of fetal macrosomia and the risk of perinatal morbidity [37]. Positive history of diabetes mellitus in a first-degree relative and multiple abnormal values in the OGTT were strongly found to predict the need for insulin management in women with GDM [38].

Studies have shown that insulin analogs (lispro and aspart) are more effective than regular human insulin in achieving targeted glucose values and minimizing the risk for macrosomia [39, 40]. There is limited data on the use of long-acting insulins in pregnancy. For women with GDM who require insulin, isophane is therefore the intermediate-acting insulin of choice [23]. Insulin analogues lispro and aspart have been widely studied and found to be clinically effective with minimal transfer across the placenta; these agents have similar safety profiles to human insulin [39]. Because the insulin analogues have shorter durations of action and more rapid onsets of action than regular insulin, they are associated with improved postprandial glycaemic control and less postprandial hypoglycaemia [41]. Glucose values that necessitate initiation of insulin are summarized in Table 3.

**Table 3:** Glucose level cut-off points requiring insulin initiation in gestation diabetes mellitus

Guideline	Fasting (mg/dl [mmol/l])	1-h postprandial (mg/dl [mmol/l])	2-h postprandial (mg/dl [mmol/l])
ACOG(22)	>95 (5.3)	>130-140 (7.2-7.8)	>120 (6.7)
ADA(15)	>90-99 (5.0-5.5)	>140 (7.8)	>120-127 (6.7-7.1)

ACOG = American College of Obstetrics and Gynecology, ADA = American Diabetes Association

#### Oral hypoglycaemics

Oral hypoglycaemic agents used in the management of GDM should be both effective and safe for the woman and developing fetus. With the exception of glyburide and metformin, oral hypoglycaemic drugs are generally not recommended due to concerns about potential teratogenicity or prolonged neonatal hypogylcaemia from drug transport across the placenta [42].

#### Glyburide

Glyburide, one of the two oral hypoglycaemic drugs used for the management of GDM, acts primarily to enhance insulin secretion by the pancreas. It can be used as an alternative for women who are unable or unwilling to take insulin or, in some cases, as a first-line pharmacological therapy. Studies have shown that glyburide, unlike other sulphonylureas, does not cross the placenta in vivo or in vitro [43, 44].

Studies examining the use of glyburide and insulin for the management of GDM have found comparative maternal and neonatal outcomes [45, 46]. Regarding glyburide therapy, certain factors are associated with higher rates of success, including initiation after 30 weeks gestation or fasting blood glucose levels <110 mg/dl and 1-h postprandial glucose levels <140 mg/dl [47]. Despite several studies supporting the efficacy and safety of glyburide for women with GDM, ACOG and ADA guidelines do not recommend its use until larger randomized controlled trials are completed on the subject [15, 22]. However, a survey conducted by ACOG found that up to 13 % of American fellows prescribe glyburide as a first-line pharmacological agent in women with GDM [48].

#### Metformin

Metformin is another oral hypoglycaemic agent considered a potential substitute for insulin in GDM management. In a randomized controlled trial involving women with GDM, the use of metformin, whether alone or with supplemental insulin, was not associated with increased perinatal complications compared to insulin alone [49]. Meanwhile, a 2013 meta-analysis found that metformin is comparable to insulin regarding glycaemic control and neonatal outcomes [50]. In another recent study, metformin use was associated with similar desirable outcomes when compared to MNT and insulin use; its use was not associated with a higher risk of maternal or neonatal complications [51].

### Glucose monitoring

In patients requiring insulin, the ideal frequency for glucose monitoring has not been established. In common practice, the patient generally checks glucose levels four times a day [23]: once upon waking in the morning, before meals, before bed and one or two hours postprandially to ensure adequate glycaemic control. Postprandial glucose levels are preferable to fasting glucose levels, because they are more strongly associated with macrosomia [52]. Insulin dose adjustments based on postprandial glucose levels rather than preprandial levels were shown to be associated with improvement in glycaemic control and reduction of both maternal and fetal adverse outcomes [53].

In ill patient with DM and other comorbid conditions, Sliding Scale Insulin (SSI) is recommended to maintain tight gycaemic control and avoid gycaemic events (i.e. hypoglycaemia and hyperglycaemia) [54]. The sliding scale insulin regimen consists of short acting insulin 4 to 6 times a day based on regularly obtained capillary blood glucose measurements. However, studies have noted that use of SSI regimen is not improving glucose control in hospitalized patient [55-58]. In addition there is no standard SSI regimen and dosage vary widely between patients, providers and institutions [59].

For women with diet-controlled GDM, there are no clinical guidelines or controlled trials addressing the issue of monitoring frequency. In this case, the general practice involves checking levels four times per day at least two days per week [23]; when two values exceed the limits over the course of a week, pharmacotherapy is recommended.

Urine glucose monitoring is not useful in patients with GDM. However, urine ketone monitoring can be used in patients who are restricting calories to detect insufficient caloric or carbohydrate intake [15].

### Intrapartum management

During labor, women on pharmacological therapy require hourly evaluations of their glucose values, while those with diet-controlled GDM do not require active glucose management. Patients on insulin usually have normal levels of glucose at the time of labor and also do not need active management [23].

### Delivery

There is no definitive data on the timing and mode of delivery for pregnant women with GDM. If the patient has normal or near normal glucose values, it is recommended that she should deliver at term. The general recommendation is that pregnancies complicated by GDM should not extend beyond term. Elective cesarean section has not been associated with significant reduction of birth trauma and has not been found to be cost effective [60]. Earlier delivery was associated with reduction of macrosomia but not with reduction of other neonatal complications [61].

### Postpartum management

After delivery, insulin resistance usually resolves quickly, as does the need for pharmacological management. However, approximately 40–60 % of affected women will develop type 2 DM later in life. They are also at an increased risk of recurrent GDM that presents earlier in future pregnancies. In these women, regular screening for type 2 DM is strongly encouraged, beginning at 6 weeks post-delivery and annually thereafter. An OGTT should be performed postpartum, 1 year post-delivery, and every 3 years thereafter [23].

## Conclusion

Despite GDM being one of the most common conditions during pregnancy, the lack of data from well-designed studies leaves some uncertainty surrounding the need for screening and management of this condition. Because the condition is associated with both maternal and fetal complications, screening and managing women at appropriate gestational age is important to minimize adverse outcomes. Glycaemic control can safely be achieved with a combination of nutritional and pharmaceutical interventions. Metformin and Glyburide have been shown to be as effective as insulin in management of GDM. Effective communication between physician, patient and primary care provider is essential, as patients experience increased rates of GDM in subsequent pregnancies and a higher lifetime risk of developing non-gestational diabetes. Further studies are required to clarify the remaining controversies surrounding diagnosis and nuanced management practices.
